# The Metabolic Activity of Caudate and Prefrontal Cortex Negatively Correlates with the Severity of Idiopathic Parkinson’s Disease

**DOI:** 10.14336/AD.2018.0814

**Published:** 2019-08-01

**Authors:** Jun-Sheng Chu, Ting-Hong Liu, Kai-Liang Wang, Chun-Lei Han, Yun-Peng Liu, Shimabukuro Michitomo, Jian-Guo Zhang, Tie Fang, Fan-Gang Meng

**Affiliations:** ^1^Department of Neurosurgery, Beijing Tiantan Hospital, Capital Medical University, Beijing, China; ^2^Beijing Neurosurgical Institute, Capital Medical University, Beijing, China; ^3^Beijing Key Laboratory of Neurostimulation, Beijing, China; ^4^Department of Neurosurgery, Beijing Children’s hospital, Capital Medical University, Beijing, China

**Keywords:** Parkinson's disease, Statistical Parametric Mapping, 18F-FDG PET, metabolic activity

## Abstract

Positron emission tomography (PET) scan with tracer [^18^F]-fluorodeoxy-glucose (^18^F-FDG) is widely used to measure the glucose metabolism in neurodegenerative disease such as Idiopathic Parkinson’s disease (IPD). Previous studies using ^18^F-FDG PET mainly focused on the motor or non-motor symptoms but not the severity of IPD. In this study, we aimed to determine the metabolic patterns of ^18^F-FDG in different stages of IPD defined by Hoehn and Yahr rating scale (H-Y rating scale) and to identify regions in the brain that play critical roles in disease progression. Fifty IPD patients were included in this study. They were 29 men and 21 women (mean±SD, age 57.7±11.1 years, disease duration 4.0±3.8 years, H-Y 2.2±1.1). Twenty healthy individuals were included as normal controls. Following ^18^F-FDG PET scan, image analysis was performed using Statistical Parametric Mapping (SPM) and Resting-State fMRI Data Analysis Toolkit (REST). The metabolic feature of IPD and regions-of-interests (ROIs) were determined. Correlation analysis between ROIs and H-Y stage was performed. SPM analysis demonstrated a significant hypometabolic activity in bilateral putamen, caudate and anterior cingulate as well as left parietal lobe, prefrontal cortex in IPD patients. In contrast, hypermetabolism was observed in the cerebellum and vermis. There was a negative correlation (*p*=0.007, *r*=-0.412) between H-Y stage and caudate metabolic activity. Moreover, the prefrontal area also showed a negative correlation with H-Y (*P*=0.033, *r*=-0.334). Thus, the uptake of FDG in caudate and prefrontal cortex can potentially be used as a surrogate marker to evaluate the severity of IPD.

Idiopathic Parkinson’s disease (IPD) is a age-related neurodegenerative disorder characterized by the asymmetrical loss of melanin-containing dopaminergic neurons in the substantia nigra [1]. Neuronal death often occurs years before the time of symptom onset in IPD. By the time a patient presents with clinical symptoms, approximately 75% of pigmented neurons in the substantia nigra (SN) were lost [2]. Tracer based method to study dopamine receptor such as ^11^C- CFT is not sensitive to predict the severity of IPD [3]. The detection of Lewy body in brainstem (within the SN) or within the cortex can confirm the diagnosis of IPD but this method is not convenient, and it needs an invasive biopsy. Currently, the diagnosis of IPD mostly depends on the presence of motor symptoms such as rest tremor, rigidity and freezing of gait, which lacks objectivity, especially in early stages of IPD as the clinical symptoms often overlap with several ‘look-alike’ disorders [4-6]. To date, the availability of quantitative biomarkers to assess the progression of the disease objectively is limited. PET/computed tomography (PET/CT) is a hybrid in-vivo imaging technique that can track the brain pathophysiological activities in a noninvasive way in various neurological and psychiatric disorders [7, 8]. The [18F]-fluorodeoxy-glucose (^18^F-FDG) PET assessing brain metabolism has demonstrated its utility in metabolic disorders including IPD [9].

Previous studies using FDG PET to analyze IPD patients mainly focused on the motor and no-motor symptoms such as cognitive, fatigue and anxiety[9-13], not the severity of disease. In addition, insights upon the progression of IPD and compensatory alterations in other parts of brain during disease progression are still lacking. Accurate and comprehensive description of the natural course and pathophysiological activity is pivotal to the assessment IPD as disease progresses. ^18^F-FDG PET metabolic imaging analysis can measure pathological activity and has been widely used in IPD. It can be potentially used as an objective, quantifiable and stable biomarker for the diagnosis of IPD as well as the evaluation of disease progression and the response to treatment [14, 15].

In this study, we evaluated the metabolic pattern of ^18^F-FDG using PET scan in 50 IPD patients and analyzed the relationship between metabolic activity of regions of interest (ROI) and H-Y stages. We identified the regions that were associated with disease progression.

## MATERIALS AND METHODS

### Subjects

From March 2016 to November 2016, 55 IPD patients were recruited consecutively from the Department of Neurology, Beijing Tiantan Hospital, Capital Medical University, Beijing, China. All patients were fulfilled the diagnostic criteria proposed by the United Kingdom PD Society Brain Bank. All had disease stages clinically defined as " medication off " state by Hoehn and Yahr’s (H&Y) rating scale and no patients had any occupying lesions in central nervous system. Among these 55 patients, 3 had progressive supranuclear palsy and 2 had severe brain atrophy; these 5 patients were excluded in this study. In total, 50 IPD patients were included for data analysis. We also recruited 20 age-matched healthy controls (11 men and 9 women, mean ± SD, 54.6 ± 12.3 years) for comparison. They showed normal neurological examination results and had no history of any central nervous system diseases. All participants provided written informed consent.

### PET Image Acquisition

Ethical permission was obtained from Beijing Tiantan Hospital, Capital Medical University. All patients and healthy controls underwent brain ^18^F-FDG PET imaging examination. Individuals fasted for at least 6 hours before ^18^F-FDG injection and stopped taking any drugs (including oral antiparkinson medications) that could affect brain metabolism for at least 3 days prior to the scanning. The ^18^F-FDG (37 MBq/kg) was injected intravenously in awake and resting state. PET image acquisition was started 60 minutes after the injection. The PET studies were performed using a Discovery ST, GE Healthcare, Waukesha, Wisconsin, USA. Computed attenuation correction was utilized to correct the brain images for attenuation of 511-keV photons. Emission images were reconstructed in a 192 ×192 × 47 matrix with a pixel size of 1.56 ×1.56 × 3.27 in the axial direction using the ordered subset expectation maximization algorithm, with 5 iterations and 32 subsets. Images were then corrected for attenuation using the CT transmission scan. Heart rate, blood pressure and pulse oximetry were measured during the PET procedure.

### ^18^F-FDG PET Analysis

Visual Evaluation of PET Data: Brain^ 18^F-FDG PET images were visually evaluated by 3 experienced nuclear medicine physicians. Brain areas with decreased and increased metabolic activity were identified and reported after at least 2 readers reached a consensus.

SPM analysis: All images were converted from DICOM to NIFTI using MRIcon (University of South Carolina, USA). Images were then analyzed voxel-by-voxel using SPM12 (Wellcome Department of Cognitive Neurology, University College, London, UK) running on Matlab (Mathworks Inc., Sherborn, MA, USA). Firstly, PET images were spatially normalized into a common Montreal Neurological Institute (MNI) atlas anatomical space, followed by a 12-parameter affine transformation and non-linear transformation. Secondly, 2×2×2mm voxels were constructed. Normalized images were smoothed with FWHM=12mm and Isotropic Gaussian Kernel to increase the signal to noise ratio. Preprocessed PET image values were corrected to a mean value of 50 mL/dL/min by “proportional scaling” to reduce individual variation. A mask with 0.8 intensity value was used to select voxels activity and to exclude extra cranial activities. A two-sample t-test was applied between diseased and control groups. To reduce the impact of age and gender, both factors were regressed out as covariates. The P value and extended voxel size (k) were thresholded at two levels and three levels, respectively: *p*< 0.001(matched with k > 50, 100, 200 voxels corrected, respectively) and* p* < 0.01 (matched with k > 50, 100, 200 voxels corrected, respectively). In addition, we chose the smaller* p* value first since *p* value wasn’t correct with false discovery rate (FDR) or Familywise error rate (FWE), but the central hypometabolic regions had been kept unchanged among at least two voxel sizes simultaneously as described in our previous study [16].

The significant clusters were tested to see whether they were closely related to clinical characters. Then the ROIs were automatically delineated with REST (http://restfmri.net/forum/REST_V1.8) on MNI atlas anatomical space with parametric PET images. Brain regions that showed *p* < 0.001 at voxel level were considered significant and were computed and transformed into z-scores. Accordingly, a subsequent correlation analysis between the metabolic activity of ROIs and H-Y rating scale was conducted using SPSS 16.0 (SPSS Inc., Chicago, Illinois).

**Table 1:** Clinical and Demographic characteristics of 50 patients (29 men; 21 women) and 20 health control (13 men; 7 women).

Characteristics	IPD (Mean/ SD, Range)	Health control (Mean/ SD, Range)
Age, yr	57.7/11.1, (31-87)	55.6 ± 12.3 (29-75)
Disease duration, yr	4.0/3.8, (0.5-17)	-
UPDRS III	27.8/8.9, (13-45)	-
H-Y last		-
Stage 1	18	-
Stage 2	11	-
Stage 2.5	4	-
Stage 3	12	-
Stage 4	2	-
Stage 5	3	-

SD = standard deviation; H-Y= Hoehn and Yahr; UPDRS III, part III of unified Parkinson’s disease rating scale.

## RESULTS

### Patient information

There were 50 IPD patients in total (29 men and 21 women, mean±SD, age 57.7±11.1 years, disease duration 4.0±3.8 years, Hoehn and Yahr Stage 2.2±1.1). Among them, 18 patients were diagnosed as H&Y stage I, 15 patient stage II, 12 patient stage III, 2 patient stage IV and 3 patient stage V (Table 1). In terms of therapy, 41 patients had received levodopa treatment after diagnosis and the remaining 9 patients did not receive medical intervention. All clinical information was listed in Table 1. Twenty age-matched healthy individuals (13 men and 7 women, mean ± SD, age 55.6 ± 12.3 years) were recruited as controls.

### Demographic characteristics

Chi-square test and Mann-Whitney U-test were performed respectively for gender and age comparison between IPD patients and healthy controls. There was no significant difference between the demographic data of the two groups, including gender (*p* = 0.292) and age (*p* = 0.21).

### PET results and statistical Analysis

By visual analysis, 90% (45/50) IPD patients appeared normal on FDG PET, and only 10% (5/50) showed abnormal metabolic activity and 3 of them had severe IPD in stage IV and V.

**Fig 1.**
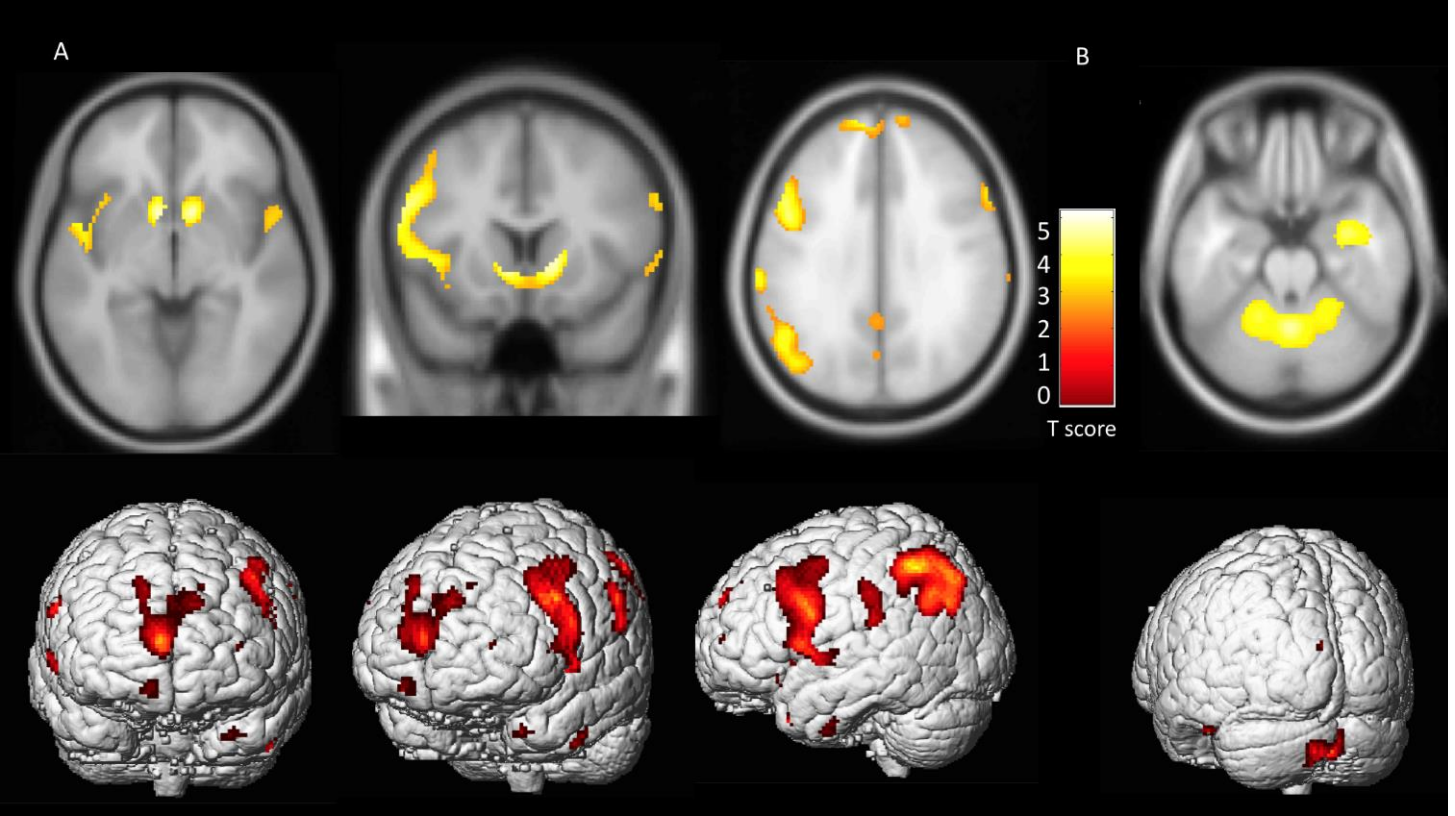
The metabolism of FDG in IPD patients compared to healthy controls. Brain areas with increased/decreased glucose metabolism are superimposed on the Montreal Neurological Institute template (Top row) (*p* < 0.001, uncorrected) and the 3D render (Bottom row). A) Significant hypometabolism in bilateral putamen, caudate, anterior cingulate, parietal lobe and prefrontal cortex was identified. B) The relative hypermetabolism was identified in the cerebellum and vermis.

Next, we performed SPM analysis. The difference between the IPD patients and the age-matched controls was calculated using the extent threshold 100 voxel-level with *p*<0.001, *P*_FDR-cor_ <0.05. As shown in Table 2, SPM analysis demonstrated that IPD patients had a significantly decreased metabolic activity in the areas of bilateral putamen, caudate and anterior cingulate when compared to heathy controls. Bilateral parietal lobes, temporal lobe and prefrontal area also showed decreased metabolic activity in IPD patients. In contrast, relative hypermetabolic activity was observed in the cerebellum and vermis in IPD patients (Fig 1).

**Figure 2.**
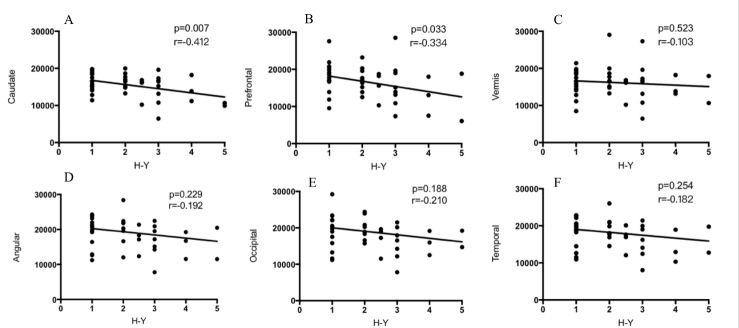
The relationship between the metabolic activity of ROIs and H-Y stages A) In caudate, the metabolic activity decreased as H-Y stages increased (*p*=0.004 r=-0.441). B) Similar to caudate, prefrontal metabolic activity also decreased as H-Y stages increased (*p*=0.004 r=-0.441). C, D, E and F, show no correlation in vermis (C), angular (D), occipital (E) and temporal lobes (F). The Pearson correlation analysis was performed using SPSS software.

To investigate the relationship between metabolic activity in significant clusters and H-Y stages, we measured the absolute glucose metabolic values in these regions and performed a correlation analysis between their metabolic activity and H-Y stages. As shown in Fig 2, caudate (A) and prefrontal (B) metabolic activity showed a negative correlation with H-Y stage, whereas no correlation was found in vermis (C), angular (D), occipital (E) and temporal lobes (F).

We also studied the relationship between disease duration and stages. H-Y stages and disease duration had a moderate positive correlation (*p* =0.001, r=0.657) and a positive linear correlation between H-Y and UPDRS III scores was identified (*p* =0.001, r=0.75).

## DISCUSSION

Molecular imaging analysis especially using ^18^F-FDG is a popular tool for the diagnosis and therapeutic evaluation of IPD. In this study we explored the metabolic activity of different brain areas in IPD patients using ^18^F-FDG PET. We demonstrated that SPM is an effective tool to analyze the data and showed that the metabolic activity of caudate and prefrontal cortex negatively correlated with the severity of disease in IPD patients.

The metabolic patterns of FDG in IPD patients have been studied but results have been inconsistent in terms of the affected areas as well as the precise distribution and the trend (i.e., increases or decreases) of metabolic changes. Eidelberg previously used PD-related pattern (PDRP) and identified increased metabolic activity in pallido-thalamic and pontine, and decreased activity in supplementary motor area (SMA), parietal association, and areas of premotor cortex [17]. The increased regions of PDRP such as the putamen and thalamus had a linear relationship with patients’ standardized motor ratings [18-20]. PD-related cognitive pattern (PDCP) was related to memory and executive function in IPD patients. It was characterized by a metabolic decrease in the medial frontal and parietal associated regions as well as a relative metabolic increase in the cerebellar vermis [21]. Other studies showed a symmetric/asymmetric hypometabolic activity in the prefrontal, lateral frontal cortices, middle temporal gyrus, bilateral parietal association cortices, and bilateral occipital cortices, as well as a relative hypermetabolic activity in the vermis and cerebellum [22, 23]. In addition, Strafella et al. found that IPD patients often showed asymmetric involvement of dopaminergic neurons in striatum [24].

In this study we found that IPD patients had reduced uptake in bilateral putamen, caudate, parietal lobe, temporal lobe, prefrontal cortex and increased uptake in vermis and cerebellum when compared to healthy controls. This is similar to the finding reported previously [23]. More interestingly, we found that there was a negative relationship between H-Y rating scale and caudate metabolic activity; the higher the H-Y scale, the lower rate of caudate metabolism. The implication of this finding is twofold: first, the measurement of caudate metabolic activity can potentially offer valuable clues for early diagnosis of IPD and monitoring disease progression; second, caudate may play an important role in the pathogenesis of IPD. Future studies to explore the role of caudate in IPD will shed light on the mechanism of disease development and progression. Of note, the potential role of caudate in IPD has been indicated in previous studies [25-28]. Autopsy study demonstrated an uneven pattern of dopamine loss in the caudate nucleus in IPD patients and previous studies showed caudate hypofunction was specific in the executive domain and related to injured nigrostriatal dopaminergic function [29, 30]. Ko et al found that the increased uptake of caudate was correlated with motor symptom severity [31]. Evidences from in vivo PET also connected caudate dopamine depletion and executive deficits in PD patients [26, 32, 33].

**Table 2:** MNI coordinate of significant clusters.

	Region	MNI coordinate			*T score*	*p*-value
		x	y	z		
IPD<HC	Caudate	12	10	0	4.46	0.000
	Frontal lobe	6	62	20	3.63	0.000
	Temporal	50	-2	-7	4.48	0.001
	Occipital	-20	-81	34	4.04	0.000
	Anterior Cingulate	10	28	22	5.00	0.001
	Parietal lobe	-50	-56	44	3.88	0.000
IPD>HC	Cerebelum	10	-62	-32	3.63	0.000
	Vermis	-32	-62	52	4.44	0.000

*P*<0.001, uncorrected; HC; healthy control; IPD: idiopathic Parkinson disease; MNI, Montreal Neurological Institute.

Another finding in our study is that prefrontal metabolic activity decreased as the disease progressed (Fig 2B, *p*=0.033, *r*=-0.334). Prefrontal cortex receives input from anterior cingulate area, a major locus of dopaminergic input to the cerebral cortex [34]. Previous study showed that cognitive decline and behavioral abnormalities were associated with functional deficits in prefrontal cortex and striatum. After deep brain stimulation of the subthalamic nucleus, the ^18^F-FDG activity in frontal lobe and the cognitive outcome showed a linear relationship. These indicated a potential relationship between striatum and frontal cortex [35, 36]. Evidences from human and monkey studies showed that caudate deficit may be related to the impaired frontal tasks [36]. In our study, both the caudate and prefrontal cortex showed a correlation to the disease severity. This indicates there may be an association between cortical and subcortical changes during IPD disease progression.

Future studies will aim to recruit more patients at stages IV and V as the majority of patients in the current study were stages I-III. Cognitive data will be collected in future studies. Other PET tracers, such as ¹¹C, H_2_^15^O will be used to determine the pattern and compare to ^18^F-FDG used in this study. Finally, a longer follow-up with changes in PET pattern will be performed for the current patient group to determine the dynamic changes in IPD.

In summary, a voxel-by-voxel based statistical mapping method, i.e. SPM12 analysis by PET scan, is a valuable tool for evaluation of disease progression in IPD. The uptake of FDG in caudate and prefrontal cortex is associated with different stages of IPD, serving as a valuable biomarker to estimate the severity of IPD.

## References

[1] Jankovic J (2008). Parkinson's disease: clinical features and diagnosis. J Neurol Neurosurg Psychiatry, 79: 368-376. 18344392 10.1136/jnnp.2007.131045

[2] Ma SY, Roytta M, Rinne JO, Collan Y, Rinne UK (1995). Single section and disector counts in evaluating neuronal loss from the substantia nigra in patients with Parkinson's disease. Neuropathol Appl Neurobiol, 21: 341-343. 7494602 10.1111/j.1365-2990.1995.tb01068.x

[3] Bernheimer H, Birkmayer W, Hornykiewicz O, Jellinger K, Seitelberger F (1973). Brain dopamine and the syndromes of Parkinson and Huntington. Clinical, morphological and neurochemical correlations. J Neurol Sci, 20: 415-455. 4272516 10.1016/0022-510x(73)90175-5

[4] Delval A, Tard C, Defebvre L (2014). Why we should study gait initiation in Parkinson's disease. Neurophysiol Clin, 44: 69-76. 24502907 10.1016/j.neucli.2013.10.127

[5] Poston KL, Eidelberg D (2010). FDG PET in the Evaluation of Parkinson's Disease. PET Clin, 5: 55-64. 20689674 10.1016/j.cpet.2009.12.004 PMC2913894

[6] Huang C, Mattis P, Perrine K, Brown N, Dhawan V, Eidelberg D (2008). Metabolic abnormalities associated with mild cognitive impairment in Parkinson disease. Neurology, 70: 1470-1477. 18367705 10.1212/01.wnl.0000304050.05332.9c PMC4454398

[7] Hellwig S, Frings L, Amtage F, Buchert R, Spehl TS, Rijntjes M, et al (2015). 18F-FDG PET Is an Early Predictor of Overall Survival in Suspected Atypical Parkinsonism. J Nucl Med, 56: 1541-1546. 26229141 10.2967/jnumed.115.159822

[8] Wang R, Xu B, Guo Z, Chen T, Zhang J, Chen Y, et al (2017). Suite PET/CT neuroimaging for the diagnosis of Parkinson's disease: statistical parametric mapping analysis. Nucl Med Commun, 38: 164-169. 27893588 10.1097/MNM.0000000000000622

[9] Wang X, Zhang J, Yuan Y, Li T, Zhang L, Ding J, et al (2017). Cerebral metabolic change in Parkinson's disease patients with anxiety: A FDG-PET study. Neurosci Lett, 653: 202-207. 28579485 10.1016/j.neulet.2017.05.062

[10] Tomse P, Jensterle L, Grmek M, Zaletel K, Pirtosek Z, Dhawan V, et al (2017). Abnormal metabolic brain network associated with Parkinson's disease: replication on a new European sample. Neuroradiology, 59: 507-515. 28386687 10.1007/s00234-017-1821-3

[11] Firbank MJ, Yarnall AJ, Lawson RA, Duncan GW, Khoo TK, Petrides GS, et al (2017). Cerebral glucose metabolism and cognition in newly diagnosed Parkinson's disease: ICICLE-PD study. J Neurol Neurosurg Psychiatry, 88: 310-316. 28315844 10.1136/jnnp-2016-313918

[12] Cho SS, Aminian K, Li C, Lang AE, Houle S, Strafella AP (2017). Fatigue in Parkinson's disease: The contribution of cerebral metabolic changes. Hum Brain Mapp, 38: 283-292. 27571419 10.1002/hbm.23360 PMC6867035

[13] Huang C, Ravdin LD, Nirenberg MJ, Piboolnurak P, Severt L, Maniscalco JS, et al (2013). Neuroimaging markers of motor and nonmotor features of Parkinson's disease: an 18f fluorodeoxyglucose positron emission computed tomography study. Dement Geriatr Cogn Disord, 35: 183-196. 23445555 10.1159/000345987

[14] Pilotto A, Premi E, Paola Caminiti S, Presotto L, Turrone R, Alberici A, et al (2018). Single-subject SPM FDG-PET patterns predict risk of dementia progression in Parkinson disease. Neurology, 90: e1029-e1037. 29453242 10.1212/WNL.0000000000005161

[15] Teune LK, Renken RJ, de Jong BM, Willemsen AT, van Osch MJ, Roerdink JB, et al (2014). Parkinson's disease-related perfusion and glucose metabolic brain patterns identified with PCASL-MRI and FDG-PET imaging. Neuroimage Clin, 5: 240-244. 25068113 10.1016/j.nicl.2014.06.007 PMC4110884

[16] Wang K, Liu T, Zhao X, Xia X, Zhang K, Qiao H, et al (2016). Comparative Study of Voxel-Based Epileptic Foci Localization Accuracy between Statistical Parametric Mapping and Three-dimensional Stereotactic Surface Projection. Front Neurol, 7: 164. 27729898 10.3389/fneur.2016.00164 PMC5037321

[17] Eidelberg D (2009). Metabolic brain networks in neurodegenerative disorders: a functional imaging approach. Trends Neurosci, 32: 548-557. 19765835 10.1016/j.tins.2009.06.003 PMC2782537

[18] Lozza C, Baron JC, Eidelberg D, Mentis MJ, Carbon M, Marie RM (2004). Executive processes in Parkinson's disease: FDG-PET and network analysis. Hum Brain Mapp, 22: 236-245. 15195290 10.1002/hbm.20033 PMC6871801

[19] Eckert T, Van Laere K, Tang C, Lewis DE, Edwards C, Santens P, et al (2007). Quantification of Parkinson's disease-related network expression with ECD SPECT. Eur J Nucl Med Mol Imaging, 34: 496-501. 17096095 10.1007/s00259-006-0261-9

[20] Asanuma K, Tang C, Ma Y, Dhawan V, Mattis P, Edwards C, et al (2006). Network modulation in the treatment of Parkinson's disease. Brain, 129: 2667-2678. 16844713 10.1093/brain/awl162 PMC4459513

[21] Huang C, Mattis P, Tang C, Perrine K, Carbon M, Eidelberg D (2007). Metabolic brain networks associated with cognitive function in Parkinson's disease. Neuroimage, 34: 714-723. 17113310 10.1016/j.neuroimage.2006.09.003 PMC4456012

[22] Juh R, Pae CU, Lee CU, Yang D, Chung Y, Suh T, et al (2005). Voxel based comparison of glucose metabolism in the differential diagnosis of the multiple system atrophy using statistical parametric mapping. Neurosci Res, 52: 211-219. 15927722 10.1016/j.neures.2005.03.010

[23] Juh R, Kim J, Moon D, Choe B, Suh T (2004). Different metabolic patterns analysis of Parkinsonism on the 18F-FDG PET. Eur J Radiol, 51: 223-233. 15294329 10.1016/S0720-048X(03)00214-6

[24] Strafella AP, Bohnen NI, Perlmutter JS, Eidelberg D, Pavese N, Van Eimeren T, et al (2017). Molecular imaging to track Parkinson's disease and atypical parkinsonisms: New imaging frontiers. Mov Disord, 32: 181-192. 28150432 10.1002/mds.26907

[25] Apostolova LG, Beyer M, Green AE, Hwang KS, Morra JH, Chou YY, et al (2010). Hippocampal, caudate, and ventricular changes in Parkinson's disease with and without dementia. Mov Disord, 25: 687-695. 20437538 10.1002/mds.22799 PMC3068920

[26] Dagher A, Owen AM, Boecker H, Brooks DJ (2001). The role of the striatum and hippocampus in planning: a PET activation study in Parkinson's disease. Brain, 124: 1020-1032. 11335704 10.1093/brain/124.5.1020

[27] Polito C, Berti V, Ramat S, Vanzi E, De Cristofaro MT, Pellicano G, et al (2012). Interaction of caudate dopamine depletion and brain metabolic changes with cognitive dysfunction in early Parkinson's disease. Neurobiol Aging, 33: 206 e229-239. 10.1016/j.neurobiolaging.2010.09.004 20961661

[28] Broussolle E, Dentresangle C, Landais P, Garcia-Larrea L, Pollak P, Croisile B, et al (1999). The relation of putamen and caudate nucleus 18F-Dopa uptake to motor and cognitive performances in Parkinson's disease. J Neurol Sci, 166: 141-151. 10475108 10.1016/s0022-510x(99)00127-6

[29] Kish SJ, Shannak K, Hornykiewicz O (1988). Uneven pattern of dopamine loss in the striatum of patients with idiopathic Parkinson's disease. Pathophysiologic and clinical implications. N Engl J Med, 318: 876-880. 3352672 10.1056/NEJM198804073181402

[30] Bruck A, Portin R, Lindell A, Laihinen A, Bergman J, Haaparanta M, et al (2001). Positron emission tomography shows that impaired frontal lobe functioning in Parkinson's disease is related to dopaminergic hypofunction in the caudate nucleus. Neurosci Lett, 311: 81-84. 11567783 10.1016/s0304-3940(01)02124-3

[31] Ko JH, Katako A, Aljuaid M, Goertzen AL, Borys A, Hobson DE, et al (2017). Distinct brain metabolic patterns separately associated with cognition, motor function, and aging in Parkinson's disease dementia. Neurobiol Aging, 60: 81-91. 28934619 10.1016/j.neurobiolaging.2017.08.020

[32] Son HJ, Jeong YJ, Yoon HJ, Kim JW, Choi GE, Park JH, et al (2017). Parkinson disease-related cortical and striatal cognitive patterns in dual time F-18 FP CIT: evidence for neural correlates between the caudate and the frontal lobe. Q J Nucl Med Mol Imaging. 10.23736/S1824-4785.17.02976-4 28750492

[33] Collins P, Wilkinson LS, Everitt BJ, Robbins TW, Roberts AC (2000). The effect of dopamine depletion from the caudate nucleus of the common marmoset (Callithrix jacchus) on tests of prefrontal cognitive function. Behav Neurosci, 114: 3-17. 10718258 10.1037//0735-7044.114.1.3

[34] Argyelan M, Carbon M, Ghilardi MF, Feigin A, Mattis P, Tang C, et al (2008). Dopaminergic suppression of brain deactivation responses during sequence learning. J Neurosci, 28: 10687-10695. 18923044 10.1523/JNEUROSCI.2933-08.2008 PMC4617653

[35] Kalbe E, Voges J, Weber T, Haarer M, Baudrexel S, Klein JC, et al (2009). Frontal FDG-PET activity correlates with cognitive outcome after STN-DBS in Parkinson disease. Neurology, 72: 42-49. 19122029 10.1212/01.wnl.0000338536.31388.f0

[36] Marie RM, Barre L, Dupuy B, Viader F, Defer G, Baron JC (1999). Relationships between striatal dopamine denervation and frontal executive tests in Parkinson's disease. Neurosci Lett, 260: 77-80. 10025703 10.1016/s0304-3940(98)00928-8

